# CAM: a novel aid system to analyse the coloration quality of thick blood smears using image processing and machine learning techniques

**DOI:** 10.1186/s12936-024-05025-7

**Published:** 2024-10-07

**Authors:** W. M. Fong Amaris, Daniel R. Suárez, Liliana J. Cortés-Cortés, Carol Martinez

**Affiliations:** 1 Pontificia Universidad Javeriana, Faculty of Engineering, Bogotá, Colombia; 2 Universidade Federal do Pará, Institute of Biological Sciences, Belém, Brazil; 3 Facultad de Ingeniería, Pontificia Universidad Javeriana, Bogotá, Colombia; 4 Laboratory of Parasitology, National Health Institute of Colombia, Bogotá, Colombia; 5 Space Robotics (SpaceR) Research Group, Interdisciplinary Centre for Security, Reliability, and Trust (SnT), University of Luxembourg, Luxembourg, Luxembourg

**Keywords:** Thick blood smears, Coloration quality, Image processing, Machine learning, Malaria diagnosis

## Abstract

**Background:**

Battling malaria’s morbidity and mortality rates demands innovative methods related to malaria diagnosis. Thick blood smears (TBS) are the gold standard for diagnosing malaria, but their coloration quality is dependent on supplies and adherence to standard protocols. Machine learning has been proposed to automate diagnosis, but the impact of smear coloration on parasite detection has not yet been fully explored.

**Methods:**

To develop Coloration Analysis in Malaria (CAM), an image database containing 600 images was created. The database was randomly divided into training (70%), validation (15%), and test (15%) sets. Nineteen feature vectors were studied based on variances, correlation coefficients, and histograms (specific variables from histograms, full histograms, and principal components from the histograms). The Machine Learning Matlab Toolbox was used to select the best candidate feature vectors and machine learning classifiers. The candidate classifiers were then tuned for validation and tested to ultimately select the best one.

**Results:**

This work introduces CAM, a machine learning system designed for automatic TBS image quality analysis. The results demonstrated that the cubic SVM classifier outperformed others in classifying coloration quality in TBS, achieving a true negative rate of 95% and a true positive rate of 97%.

**Conclusions:**

An image-based approach was developed to automatically evaluate the coloration quality of TBS. This finding highlights the potential of image-based analysis to assess TBS coloration quality. CAM is intended to function as a supportive tool for analyzing the coloration quality of thick blood smears.

**Supplementary Information:**

The online version contains supplementary material available at 10.1186/s12936-024-05025-7.

## Background

Malaria is an infectious disease and one of the most critical public health problems worldwide. According to the World Health Organization (WHO), in 2022, there were 249 million malaria cases, an increase of 5 million cases compared with 2021 [1]. In Colombia, where malaria outbreaks are common in rural areas, health posts and patients must deal with geographical limitations, violence-related problems, and severe climate conditions, among other factors. These conditions affect the dynamics of rural health posts, which must streamline the process, dealing quickly with all the cases (conducting fast sample preparation and quick and accurate diagnosis) [2, 3].

The microscopic diagnostic procedure is considered the “gold standard” method worldwide, and thick blood smears (TBS) is the reference method chosen as the first option for malaria diagnosis [4–8]. TBS allows quantitative results to monitor patients by analysing the parasitaemia before, during, and after the treatment [6, 9].

Studies have been conducted to automate malaria diagnosis using images from thin and thick smears. However, regularly, those studies have limitations, such as the exclusive use of high-quality images that do not include common imperfections like the presence of image artifacts, staining troubles, and factors related to the non-standardization of the image acquisition process, for example, illumination and the type of functions used from the microscope where they acquired the photographies [10–20].

Based on the literature review, the primary focus of published works is on devising strategies to facilitate malaria diagnosis, aiming for quick and parasite detection. Many papers highlight the issues associated with inconsistencies in the staining of thin and thick smears [10–14, 17–22]. These inconsistencies are particularly prevalent in TBS, as noted by multiple studies [13, 14, 21, 23, 24]. However, an unaddressed area in the research is the analysis of the quality of smear coloration. The variations in smear coloration could potentially impact malaria diagnosis, indicating a need for further investigation into this factor.

Malaria diagnosis, involving a roughly one-hour sample collection, preparation, and visual examination using TBS, is subject to considerable variability and subjectivity [4, 25]. Influencing factors include the conditions and location of sample preparation, the availability and quality of equipment and supplies like stains, and minor changes in stain concentration or pH levels of the staining solution or washing water [26]. The staining stage is particularly critical as it directly impacts the visibility of parasites and, thus, the accuracy of diagnosis [27]. However, this stage involves a subjective estimation process where the determination of coloration depends on human judgment and the microscopists’ experience, adding another layer of subjectivity [26–29]. The latter underscores the importance of stringent quality control and standardization in malaria diagnosis to minimize these factors’ impact. Recognizing the limitations previously discussed, in this work, Coloration analysis in malaria (CAM) is a system that uses image processing and machine learning algorithms. CAM is designed to assess the colour quality of TBS and automatically verify if they meet the essential quality criteria, such as appropriate background colour, thereby improving the process of microscopic examination in malaria diagnosis.

In previous works [28, 29], it was conducted an extensive image processing analysis to determine the best features for evaluating the background coloration quality in TBS. The current paper describes the workflow of CAM and a comprehensive evaluation of machine learning techniques that are the basis for the presented analyses. A database of 600 images of *Plasmodium vivax* TBS with balanced classes (good/bad coloration quality) was created to train, validate, and test machine-learning classifiers. To discriminate the two quality classes (good or bad), this paper presents the tuning of the parameters and the selection process of the best feature vector and classifier.

## Methods

**Fig. 1:**
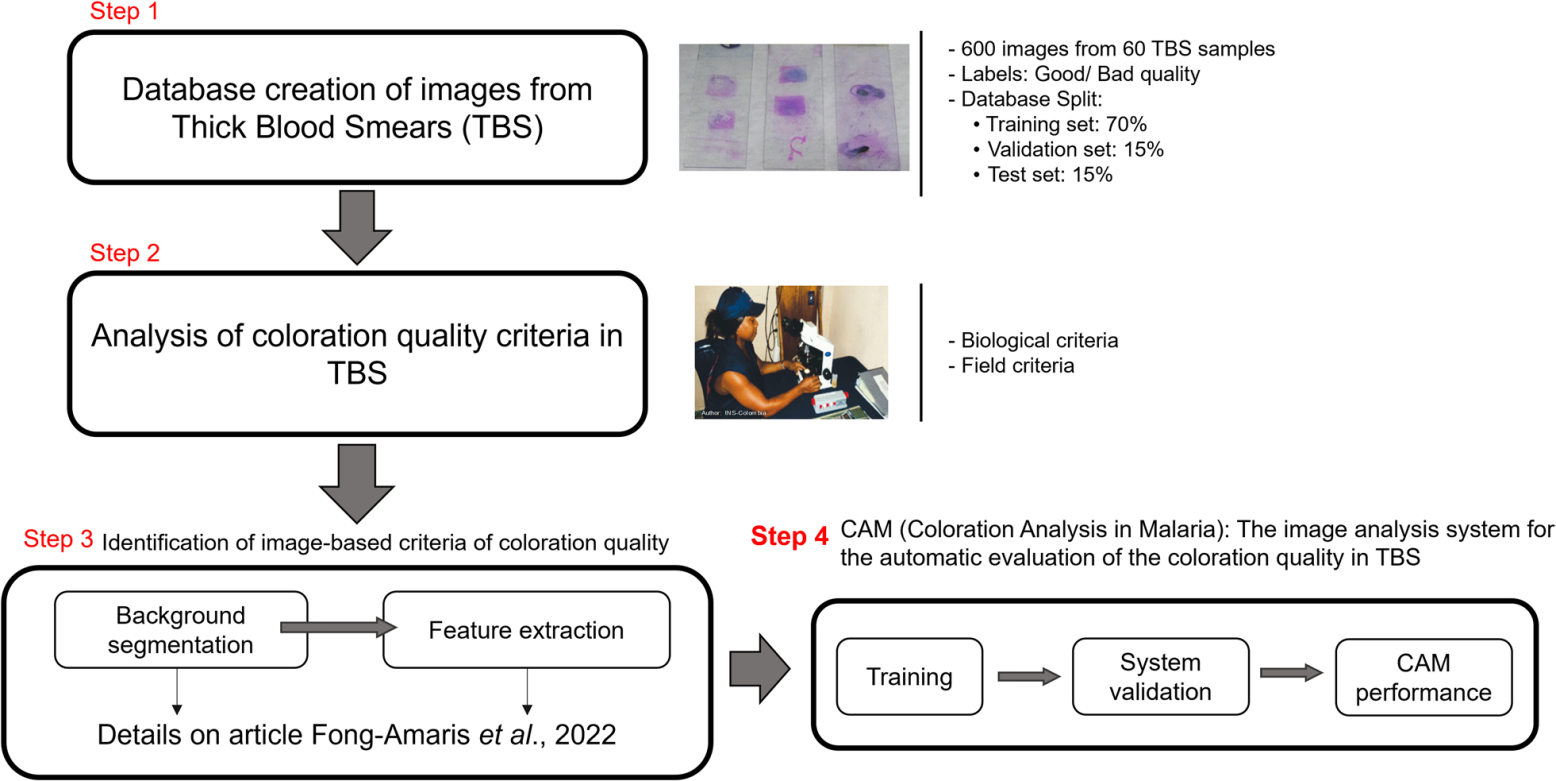
Methodological steps followed to evaluate the staining of a malaria smear by analysing the coloration quality of TBS

The four-step methodology presented in Fig. 1 was followed to develop the image-based approach to automatically evaluate the coloration quality of TBS (stained with the modified Romanowsky stain [4, 30, 31]. The first step corresponds to creating the database with images from TBS with good and bad coloration quality. The database was used to select image-based criteria of coloration quality and to establish and evaluate the machine learning classifier of CAM. The second step corresponds to the analysis of coloration quality criteria used in malaria diagnosis laboratories to assess the quality of TBS. The third step corresponds to identifying image-based criteria to evaluate the coloration quality [29]. In this step, different image-based processing techniques were tested to select the most suitable features to identify the coloration quality. Finally, the fourth step corresponds to selecting and tuning a classifier that automatically evaluates the coloration quality in thick blood smear images. Steps 1, 2, and 3 were presented and described in [29]. This paper focuses on Step 4, the methodology’s feature and classifier selection steps.

### Image database of TBS

A database containing images and labels was created to design and evaluate different image-based strategies to determine the quality of the staining procedure for TBS stained with modified Romanowsky stain. Most literature on parasite detection in TBS uses staining with Giemsa [20, 32, 33] and Jaswant Singh Battacharya stain [13]. However, to the authors’ knowledge, no study has used databases with images from TBS stained with modified Romanowsky stain. Nor are publicly available databases related to the coloration quality of TBS (good/bad).

The images were captured following a protocol to ensure that the acquired images contained the same colour information as that seen through the microscope. Details of the protocol can be found in [29]. The optical microscope (Axio Zeiss Scope.A1, Brand: Carl Zeiss, Country: Germany) was used to create the database. The 100X microscope lens used for the microscopic visualization has the reference: Objective-lens A-Plan 100×/1.25 Oil M27, Carl Zeiss, Germany.

**Fig. 2:**
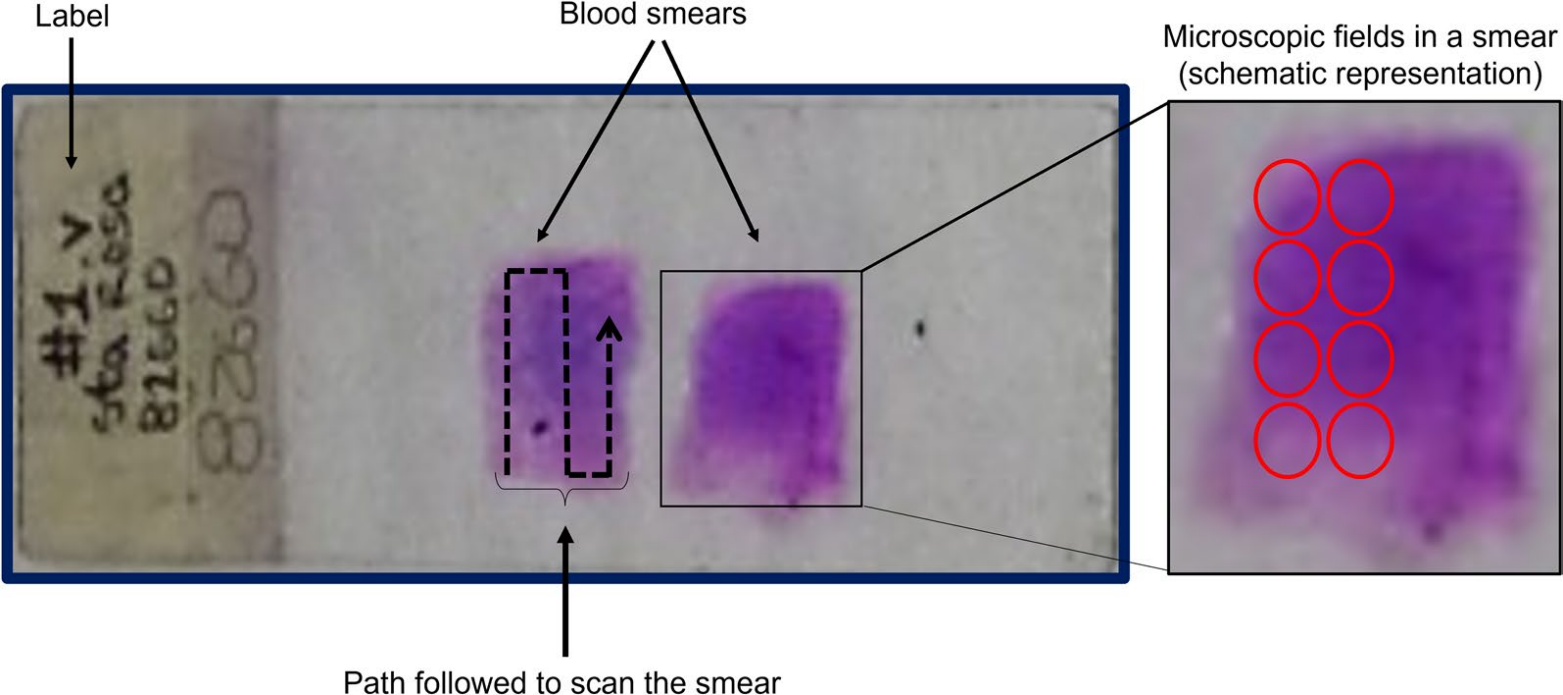
Indication about how a TBS is read when malaria is diagnosed. The two staining squares represent two blood drops spread over a slide. The left written label corresponds to smear identification. It can be numerical or descriptive. The dashed arrow shows the route of the microscopic visualization recommended by the WHO to guarantee a correct reading. The red circles indicate the schematic representation of 8 microscopic fields

The study population corresponds to malaria cases due to *P. vivax* in 2017 and 2018. *Plasmodium vivax* is the predominant parasite in Central and South America, representing 75% of malaria cases [34]. From a complete set of 60 TBS, 600 photographic records (10 images of different fields per slide) were obtained. The captured areas correspond to fields close to the sample’s centre to ensure uniformity in the images (in periphery regions, the thickness of the blood can vary), as depicted in Fig. 2. The images acquired through the optical microscope are 2056 × 2452 pixels and were saved in Portable Network Graphics (PNG) format.

The 600 images were labelled using the web-based labelling tool Labelbox [35]. Two labels were created for the background coloration quality, which can be good (1) or bad (2). The determination of TBS quality (good or bad) was defined based on the background colour of the sample as it was described in previous work [29] and according to the established by the INS-Colombia [25], WHO [4] and other works [36, 37]. If the background showed a pink or light blue colour with pink areas in the microscopic field, it was considered bad quality. Otherwise, the image was classified as having good coloration quality (Table 1). Finally, the database (600 images) was randomly split into training (70%), validation (15%), and test (15%) sets, as shown in Table 1.

**Table 1:** Database of TBS stained with the modified Romanowsky stain

Labels	Classification	Quantity	Database split		
			Training	Validation	Testing
Coloration quality	Good	300	210	45	45
	Bad	300	210	45	45

All sets contained balanced classes. The database instructions and labels can be found using the 10.5281/zenodo.7191423 [38].

#### Coloration quality criteria

Depending on the staining procedure, the staining of a thick blood smear can be of good or bad quality, and the presence of parasites can be easily detected or not.

#### Biological criteria

**Fig. 3:**
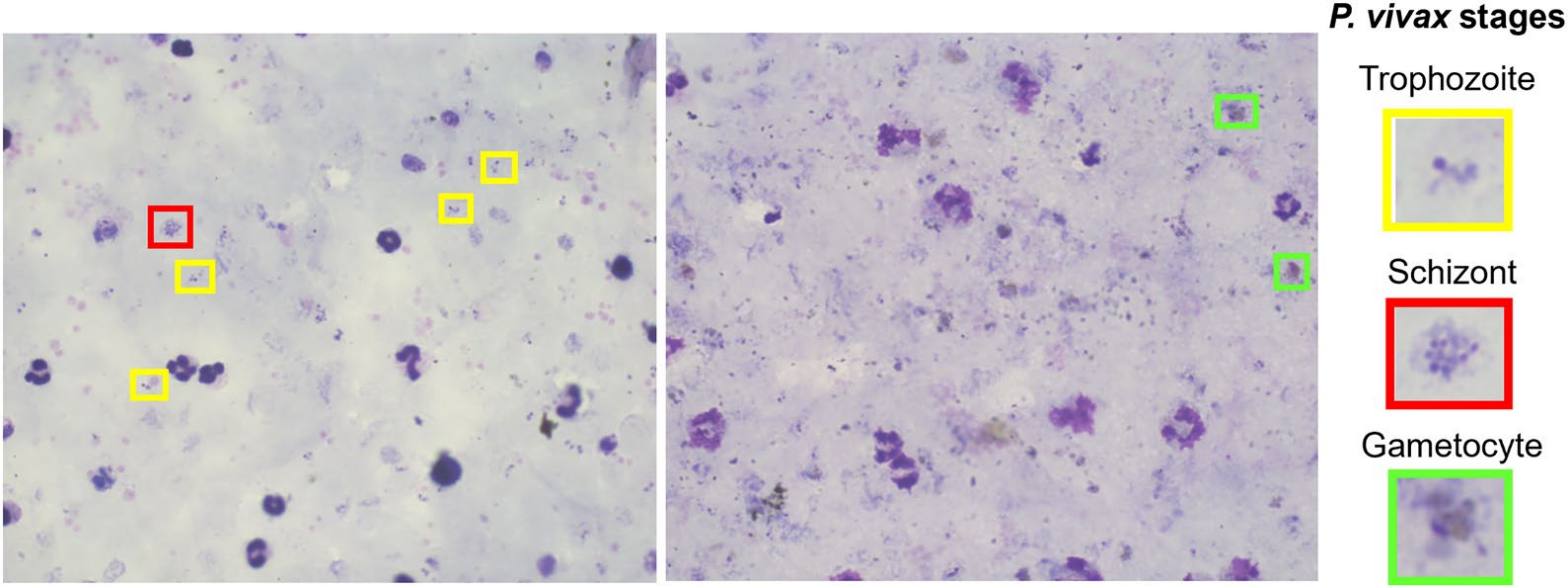
Two microscopic images obtained from TBS with good coloration quality. This figure shows examples of the life cycle stages of the parasite *P. vivax*, where the yellow, red, and green squares correspond to the trophozoite, schizont, and gametocyte stages, respectively

Factors such as the background colour of a thick smear, the colour of the nucleus and the cytoplasm from leukocytes and parasites (Fig. 3), the colour and morphology of the platelets, and minor differences in pH are indicator factors of the quality of the stain in a thick smear. They give information about how well-stained a smear is. There are more details about this section in a previous article where it was mentioned that the appearance of the smear background is one of the most critical features that indicates information about the coloration quality in a thick blood smear [29].

### Image-based criteria of quality

#### Background segmentation

The biological criteria of quality showed that the appearance of the background of the smear is a crucial feature in evaluating the quality of the coloration. Therefore, from the microscopic images of the TBS, the background information had to be separated from the foreground information (leukocytes, parasites, and platelets) to ensure that the image-based analysis was conducted only on the background information.

The microscopic image is captured in the RGB colour space. However, this image can be transformed into other colour models, called colour spaces, to extract specific features. Colour as a feature can give us valuable information to characterize the coloration in TBS.

In the literature, previous works focused on parasite detection and classification have mainly used the RGB and HSV colour spaces [10, 11, 13, 14, 18–21, 32, 39, 40]. These colour spaces have been used to segment the parasite nucleus, contrast enhancement, erythrocyte segmentation, and even background removal in images from thin blood smears [41]. HSV colour space has been used in studies related to parasite detection [13] and classification [32] to segment foreground elements such as parasites, leukocytes, and platelets. In the case of the study done by Chakrabortya et al*.* [13], they used the H and S components to extract the foreground elements. However, their TBS were stained with the Jaswant Singh Battacharya stain, which gives different colour saturation to the biological elements from the smear [13].

**Fig. 4:**
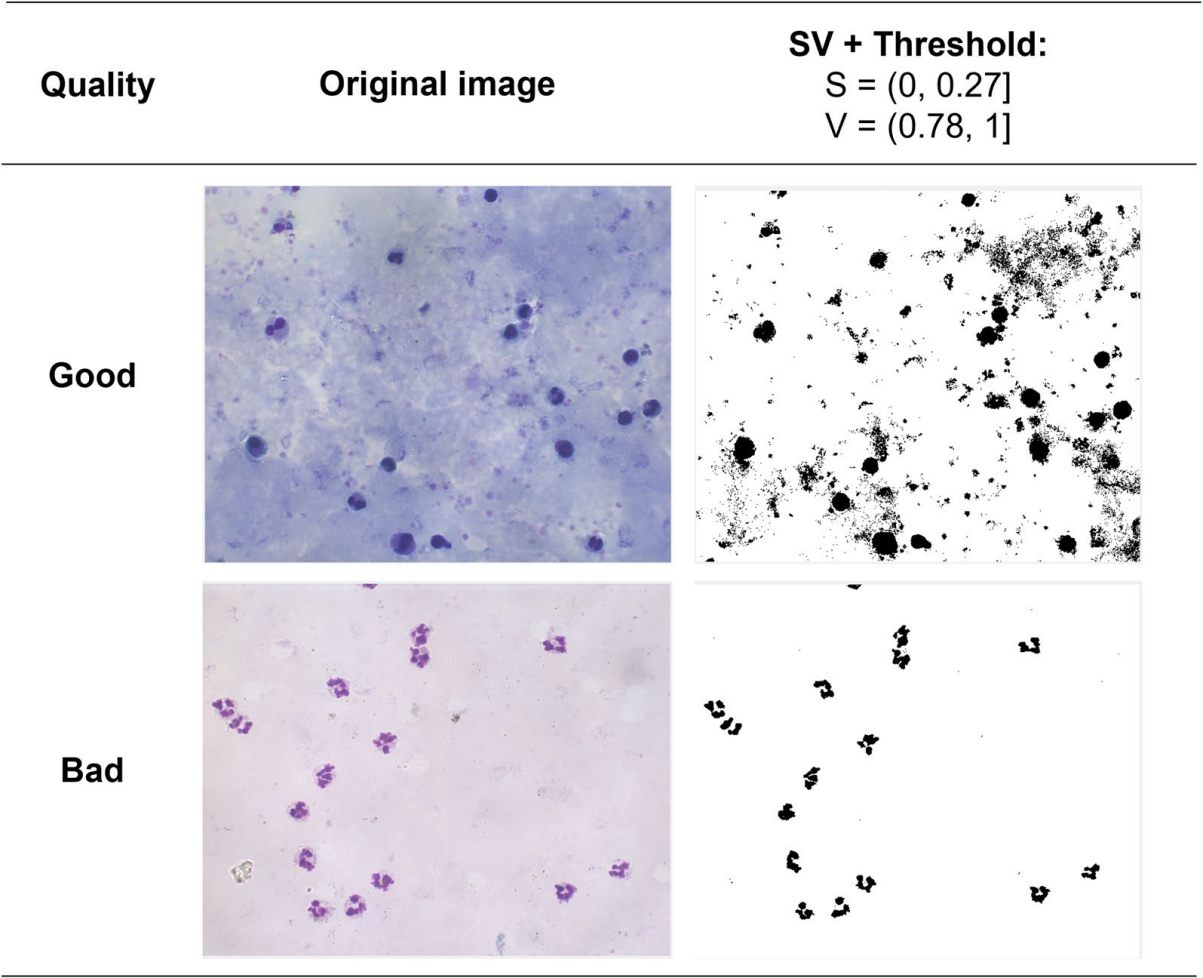
Threshold selection from the S and V combined components from the HSV colour space. SV + Threshold: the RGB image was transformed into the HSV colour space. From this image, it was obtained a threshold by each component (S and V), and the pixels that fell under the threshold were considered background; otherwise, they were foreground

Because previous work had not studied the background information as a feature for estimating the coloration quality in TBS, an exploratory analysis with different components of the HSV and RGB colour spaces (analyzed in our previous work) [28, 29] was conducted to find the best threshold and the best colour space for background segmentation. The best colour space was carefully selected, ensuring stained leukocytes were not confused as background information. The S and V components were selected as they showed similar background segmentation capability for TBS images of good and bad coloration quality. The threshold values used were S = (0, 0.27] and V = (0.78, 1] (Fig. 4).

#### Feature extraction

Computer vision techniques applied to parasite detection and classification in blood smears have been used in previous studies to respond to the need for new alternatives to reduce malaria by 2030 [42]. Combined with machine learning, image features such as colour, texture, shape, and size are commonly used for parasite detection [13, 14, 20, 21, 23, 24, 32]. However, the challenge is to include the preprocessing stages of the smears in the analysis, including varying parameters for image acquisition and slide preparation [16, 26]. Any automatic malaria diagnosis system should include those critical steps before the classification system.

Despite the few studies focused on TBS [13, 14, 20, 21, 23, 24, 32], some have used features based on histograms for malaria parasite detection, as in Hanif et al*.* [23] and Salamah et al. [24]. These studies used threshold-based segmentation and colour-based histograms, respectively. On the other hand, the machine-learning techniques for malaria diagnosis have focused on parasite detection and classification using k-means clustering and support vector machines [16]. According to previous research in the field, a histogram-based feature extraction procedure was considered one of the strategies for our work.

**Fig. 5:**
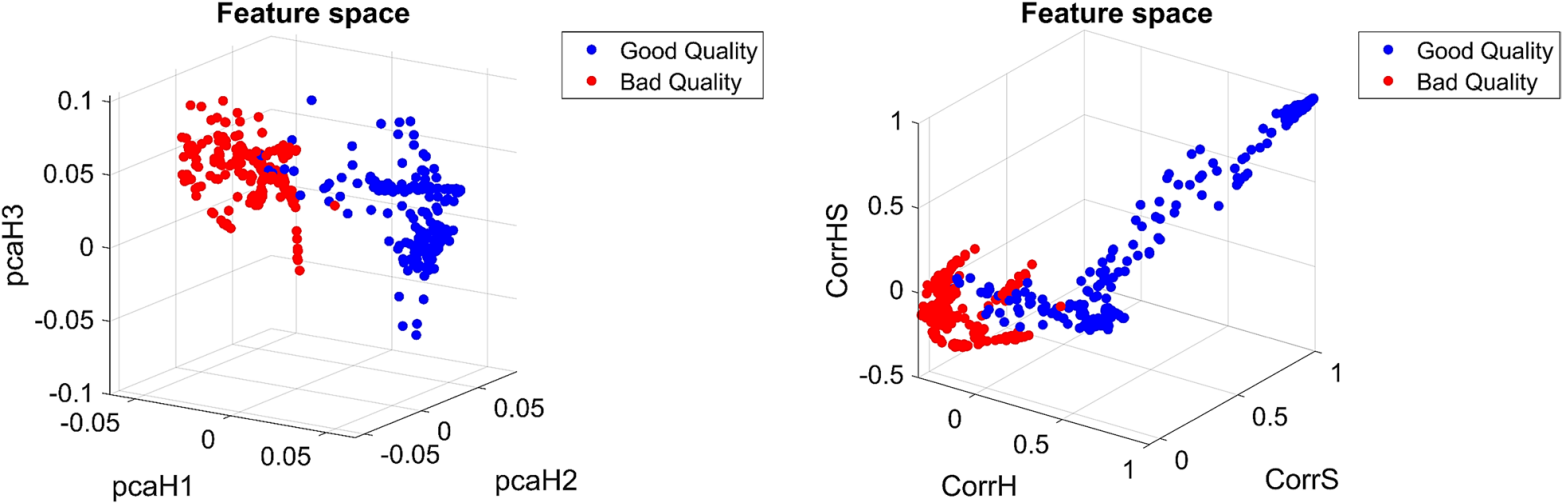
Features space for the [H_PCA1_, H_PCA2_, H_PCA3_] (at left) and [Hcorr, Scorr, HScorr] (at right) features vector. The red points represent the images with bad coloration quality, and the blue points represent the images with good coloration quality

**Fig. 6:**
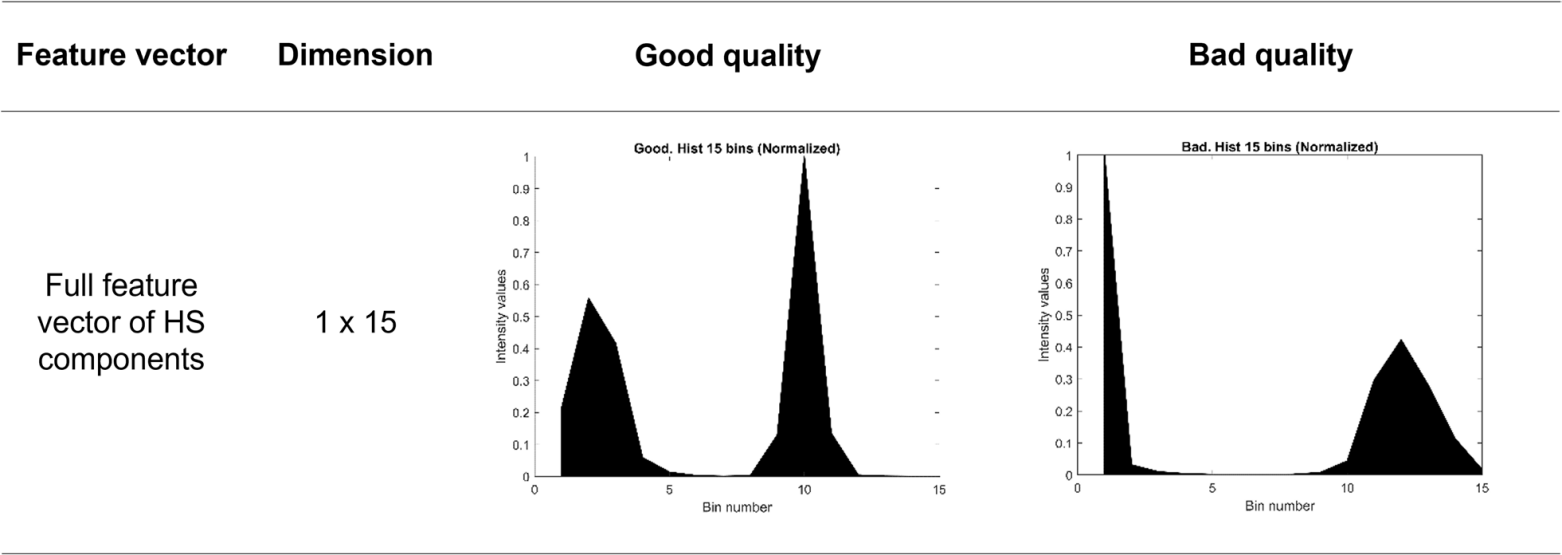
Histograms using 15 bins with HS combined components. The histograms did not include the black pixels from leukocytes. The figure shows the data distribution: the left shape in each graph corresponds to the pixel distribution for the S component, and the right shape shows the pixel distribution for the H component

The analysis of 19 feature vectors involved variances, correlation coefficients, and histograms (specific variables from histograms, full histograms, and principal components from the histograms). In previous work [29], the ability of 19 feature vectors to classify images based on good and bad coloration quality was analyzed. As a result, four feature vectors ([H_PCA1,_ H_PCA2,_ H_PCA3_], [Hcorr, Scorr, HScorr], Hist_15__H, and [H_PCA1,_ H_PCA2,_ H_PCA3_, S_PCA1_, S_PCA2_, S_PCA3_, HS_PCA1_, HS_PCA2_, HS_PCA3_]) were selected as candidates to describe the background information according to the coloration quality. Figure 5 shows the feature space of two feature vectors such as [H_PCA1,_ H_PCA2,_ and H_PCA3_] and [Hcorr, Scorr, HScorr]. The histogram obtained for Hist_15__H is shown in Fig. 6.

#### Machine learning classifiers

**Fig. 7:**
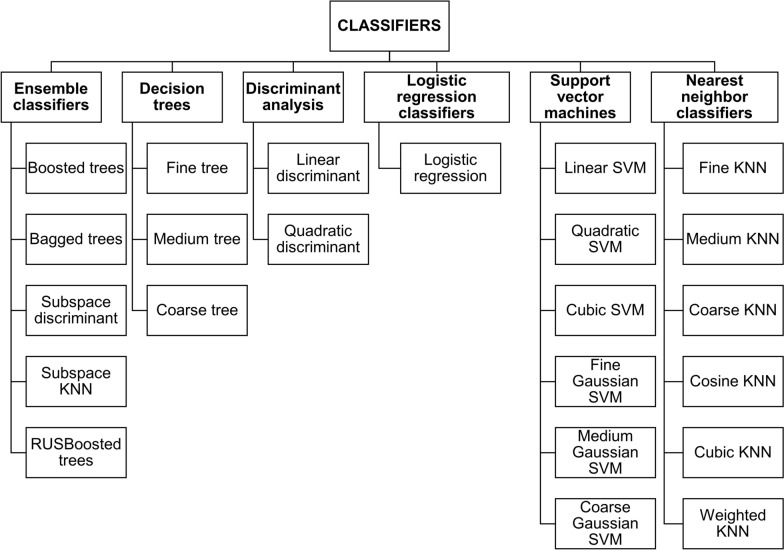
Matlab classifiers used for selecting the preliminary 4-best feature vectors

The Machine Learning Matlab Toolbox [43] was used to select the best feature vector and machine learning classifier. The 23 classifiers available in the toolbox (Fig. 7) were trained with their default parameters, using as feature vectors the ones presented in section “Feature extraction”. The training and validation set from the database (“Image database of TBS” section) were used. The True Negative Rate (TNR), the True Positive Rate (TPR), and the F1 score were analysed to evaluate the results. Nevertheless, the TNR was considered the most critical metric. To predict ‘good’ when in reality ‘bad’ is the attribute for assessing coloration quality could harm patient health. A smear that is not stained correctly can lead to errors in detecting the parasite. Therefore, it may result in errors in calculating patients’ dosage and parasite density. That is why TNR was the interest metric. This metric would facilitate the evaluation of the system’s ability to discriminate bad-quality samples.

Table S3 in Supplementary material shows the classifiers that presented the best training results in terms of the TNR and F1 score in each of the tested feature vectors (SVM Quadratic, SVM Cubic, SVM Medium Gaussian, SVM Fine Gaussian, Logistic Regression).

Since most classifiers achieved accuracy rates exceeding 95% across all candidate feature vectors (including [H_PCA1,_ H_PCA2,_ H_PCA3_], [Hcorr, Scorr, HScorr], Hist15_H, and [H_PCA1,_ H_PCA2,_ H_PCA3_, S_PCA1_, S_PCA2_, S_PCA3_, HS_PCA1_, HS_PCA2_, HS_PCA3_]), Quadratic SVM and Cubic SVM were selected for further evaluation due to their superior performance. The classifiers mentioned before were used to develop the system adjustments (Table S2 in Supplementary material).

### CAM: coloration analysis in malaria

**Fig. 8:**
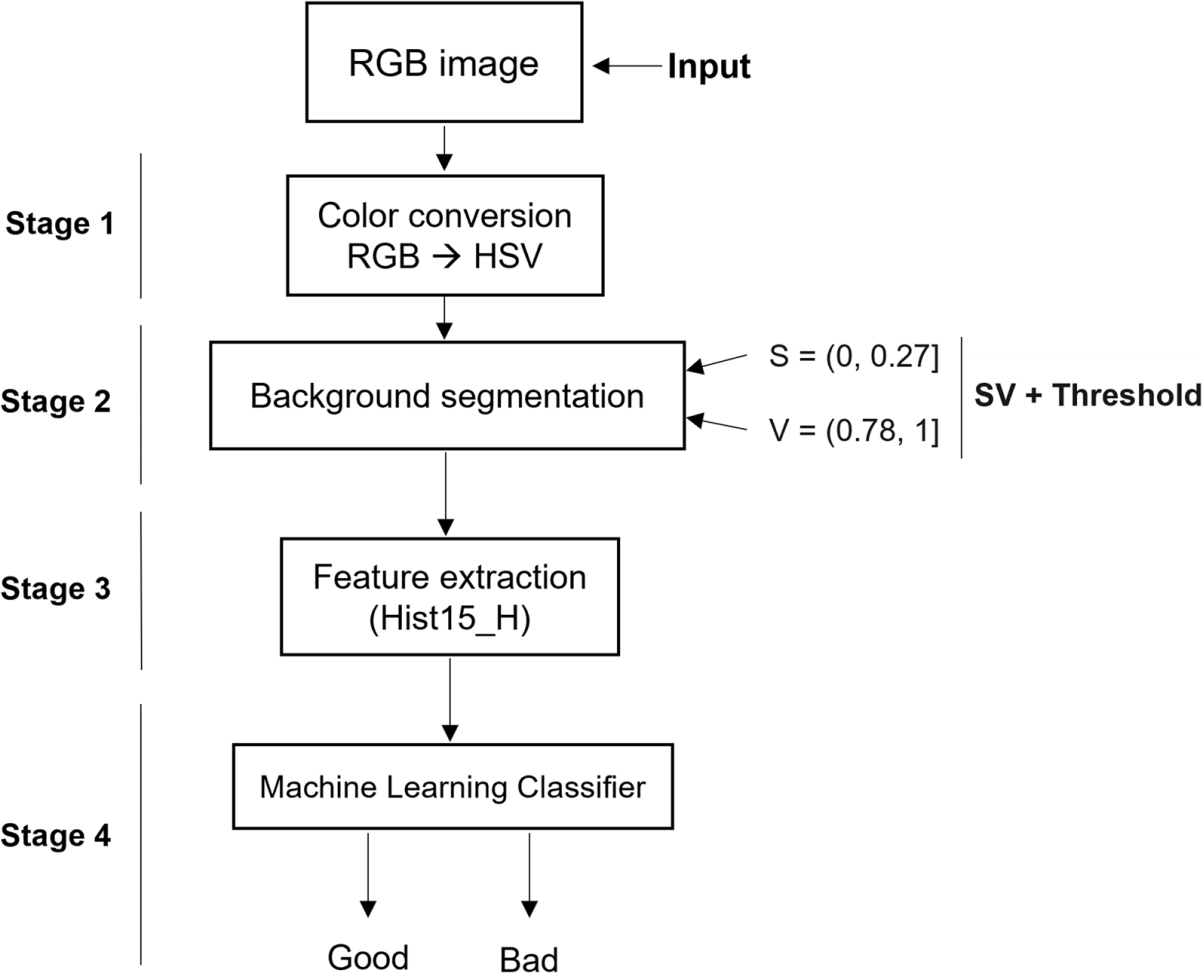
Algorithm for the automatic quality analysis of TBS in malaria called Coloration Analysis in Malaria (CAM)

CAM is an algorithm that automatically analyses the coloration quality of TBS. It was designed using MATLAB® Student, Version R2018a, Mathworks, USA [43]. The proposed algorithm consists of four stages, as presented in Fig. 8 and described below.

- Stage 1: As input, CAM receives an image in RGB format, which is then transformed into the HSV colour space.
- Stage 2: The histograms of each component of HSV were extracted. The S and V components were integrated into a histogram called SV, which was thresholded to segment the foreground elements (leucocytes and parasites) from the background, obtaining the thresholded image. To isolate the background information of interest in the HSV color space, the thresholded image was multiplied by the original HSV image, resulting in only the background information remaining and the foreground elements appearing black.
- Stage 3: This stage relates to feature extraction, as found in [28, 29], that extracting features in the HSV colour space makes it possible to find image criteria of coloration quality. The feature vector is used as input for Stage 4. With that information, two possible features can be extracted, which have been demonstrated to provide good results: the 15-bin histogram of the H channel (Hist_15__H) and the three main PCA components from the histogram of the H channel [H_PCA1,_ H_PCA2,_ H_PCA3_].
- Stage 4: A machine learning classifier is in charge of estimating the coloration quality of the image background. The algorithm’s output will be “good” if the quality of the coloration is good or bad if the background coloration quality of the image is not appropriate for diagnosis, according to the image’s criteria of quality that was presented in [28, 29].

A system like CAM can be used in isolation to evaluate the abilities of microscopists, e.g., when assessing microscopists’ smear staining capabilities, to support microscopists by making them aware that a careful analysis of each microscopic field is required, or as a subsystem of an automated malaria diagnosis system.

## Results

The first test below presents the process for selecting the best feature vector and classifier for CAM. Once selected, CAM’s performance is evaluated in terms of TNR, TPR, accuracy, misclassification rate, and F1 Score.

### Classifier optimization

The Quadratic SVM and the Cubic SVM classifiers were selected for tuning to optimize the coloration quality estimation algorithm. Code for adjustments was obtained from the Machine Learning Toolbox in MATLAB [43].

The default values for the Quadratic and Cubic SVM include a polynomial kernel function, the polynomial order (2 for Quadratic SVM and 3 for Cubic SVM), and the automatic kernel scale (default value 1). The supplementary material (Table S3) contains details about the results obtained during the optimization process of the classifiers. Table S3 summarizes the final candidate classifiers and the features and parameters that showed the best results for assessing the background quality using the validation set. According to the results, the best features that can be used to analyse the coloration quality in thick blood smear images were feature vectors 1 and 3, respectively: [H_PCA1,_ H_PCA2,_ H_PCA3_] and (Hist_15__H).

### CAM performance

The algorithm’s performance was evaluated using the test set from the database, considering both the selected classifier and the two candidate feature vectors. The obtained rates are presented in Table 2**.** Analysis of the results revealed that one of the feature vectors, Hist_15__H, exhibited superior predictive behaviour (TNR: 95%, TPR: 97%, F1-score: 98%) than the [H_PCA1,_ H_PCA2,_ H_PCA3_] feature vector (TNR: 95%, TPR: 95%, F1-score: 95%). The latter is because the values (in terms of TPR, Accuracy, and F1-score) obtained from the feature vector Hist_15__H were higher than 95%. Although the misclassification rate was lower in the feature vector [H_PCA1_, H_PCA2_, H_PCA3_], the TNR, TPR, accuracy, and F1-score rates were also considered to make the selection.

**Table 2:** Performance of the cubic SVM classifier predicting the coloration quality of the images of the test set

Features	Rates				
	Misclassif ^a^	TNR	TPR	Accuracy	F1-score
[H_PCA1,_H_PCA2,_ H_PCA3_]	0.0190	0.9556	0.9556	0.9556	0.9556
Hist_15__H	0.0214	0.9556	0.9778	0.9667	0.9888

^a^Misclassif.: out-of-sample misclassification rate

The confusion matrixes in Table 3 indicate the classifier performance obtained using the feature vectors [H_PCA1_, H_PCA2_, H_PCA3_] and [Hist_15__H].

**Table 3:** Confusion matrixes obtained for each feature vector ([H_PCA1_, H_PCA2_, H_PCA3_] and [Hist15_H]) using the cubic SVM

[H_PCA1_, H_PCA2_, H_PCA3_]			[Hist15_H]		
Total: 90	Predicted: bad quality	Predicted: good quality	Total: 90	Predicted: bad quality	Predicted: good quality
Actual: bad quality	43	2	Actual: bad quality	43	2
Actual: good quality	**2**	43	Actual: good quality	**1**	44

**Fig. 9:**
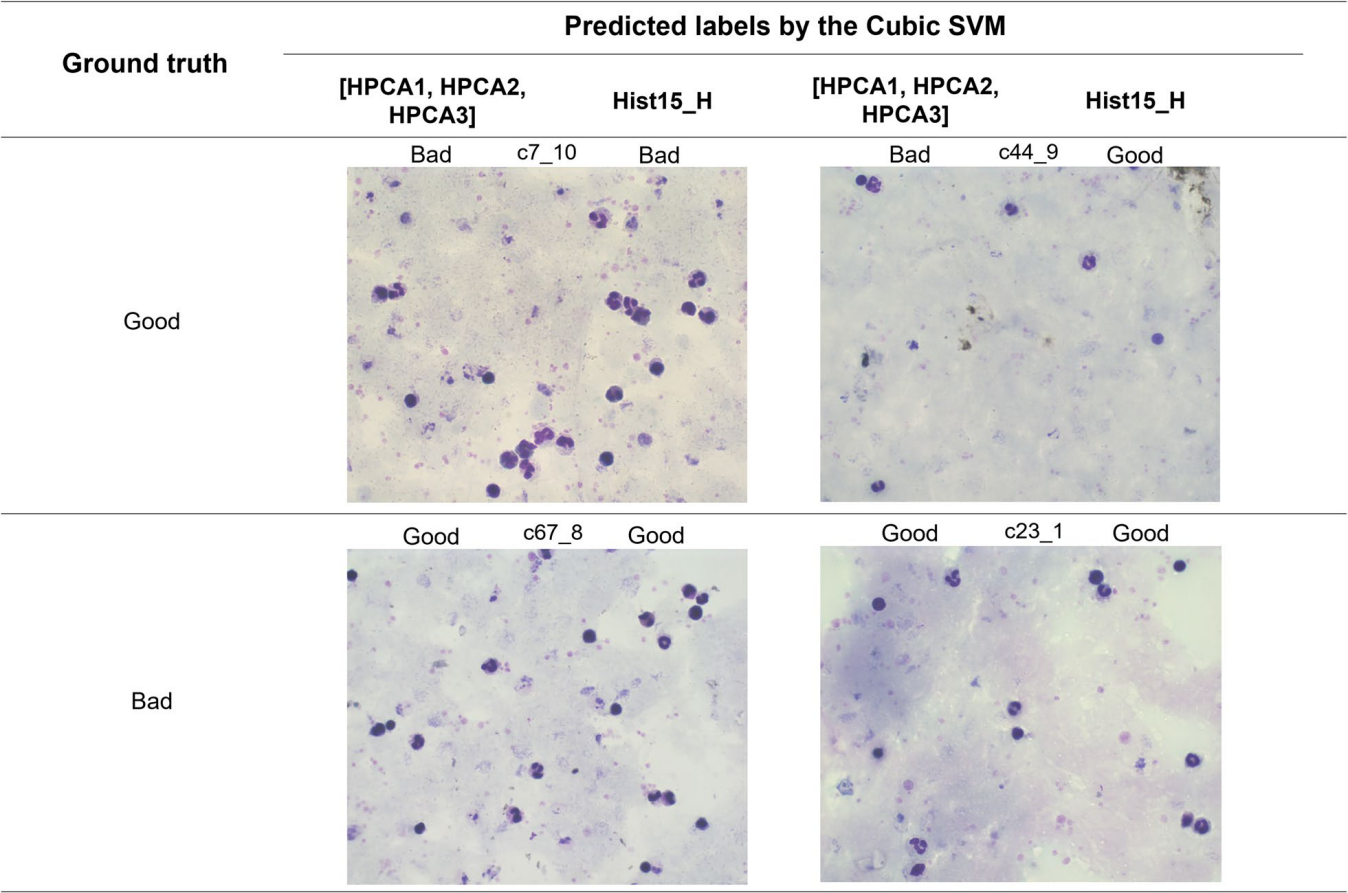
Errors by the Cubic SVM using the [H_PCA1,_ H_PCA2,_ H_PCA3_] and Hist_15__H feature vectors

Based on Table 3, the images where the algorithm did not estimate the quality correctly were analysed. Table 3 shows images corresponding to two good and bad coloration quality. For the analysis, it is essential to consider that a good background coloration quality is related to a blue colour, a homogeneous sample spreading, and other factors mentioned in [4, 25]. The images in Fig. 9 present differences in the coloration background.

Analysing those images, the wrong labels occurred with images that contained parasite presence. The images without parasites did not present that misclassification. In the images shown in Fig. 9, three presented a non-homogeneous background. This means that the blood sample was not spread adequately above the slide. Thus, the colour information is not homogeneous, which could affect the label. On the contrary, the image c44_9 (Fig. 9) showed a homogeneous background.

Analysis revealed differences in the classifier’s predictive ability for good quality images when using each feature vector. Notably, the Cubic SVM classifier exhibited one additional misclassification using the [H_PCA1,_ H_PCA2,_ H_PCA3_] feature vector compared to Hist_15__H.

Images c7_10 and c44_9 from Fig. 9 show examples of false-negative predictions according to the ground truth column. Using both feature vectors ([H_PCA1,_ H_PCA2,_ H_PCA3_] and Hist15_H), the background of the image c7_10 (left image from Fig. 9) was wrongly predicted. This image presented a no homogeneous stain distribution and a low sample thickness. The second image (c44_9, right image from Fig. 9) was only misclassified by the feature vector [H_PCA1,_ H_PCA2,_ H_PCA3_]. This image presents a pale blue colour in the background and a sample spreading of type homogeneous. The higher bin number of the feature vector Hist15_H than the feature vector [H_PCA1,_ H_PCA2,_ H_PCA3_] allows for distinguishing different colour tonalities, which helps correct the image classification.

On the other hand, in the images with bad coloration quality, the classifier with the two different feature vectors misclassified the same images (c67_8 and c23_1). Both images present a pale blue colour in the background (blue background is characteristic of good coloration quality). However, the images show a non-homogeneous colour distribution with pink colour areas related to the misclassification in both feature vectors (there were areas without blood-stained samples).

The results described above about the predictive capability of the feature vectors suggest that the Hist_15__H feature vector is the best vector for describing and classifying the background coloration quality in TBS stained with modified Romanowsky stain.

Thus, the feature vector Hist_15__H and the Cubic SVM classifiers were selected to be part of CAM.

## Discussion

Currently, available literature has focused on methods to support malaria diagnosis. Previous studies have noted that the coloration quality of images obtained from TBS complicates the comparison of studies involved in automatic parasite detection. Despite achieving reasonable sensitivity and specificity rates, the different systems developed so far cannot be compared due to differences in coloration quality [16, 44].

This paper presents CAM, the first algorithm proposed to automate the analysis of coloration quality in TBS. This is the first report on using an SVM classifier in studies that analyse TBS coloration quality for malaria diagnosis.

According to Table 2, the algorithm achieves a True Positive Rate (TPR) of 97% in predicting the background quality in images with good coloration. The best results for predicting coloration quality were obtained with the Hist_15__H feature vector, corresponding to the 15-bin histogram of the Hue channel from the HSV colour space. The following evidence supports the previously reported findings [28, 29]. The study represents a pioneering step towards analysing image quality, providing a basis for comparing studies by considering the number of images with superior and inferior coloration quality and their respective performance outcomes.

This study’s findings offer initial steps towards automating the assessment of coloration quality procedures in Departmental Laboratories of Public Health to ensure effective diagnostics. Thus, CAM is introduced as a novel aid system for analysing the coloration quality of thick blood smears using image processing and machine learning techniques.

While CAM represents a significant advancement in automating the analysis of coloration quality in thick blood smears (TBS) for malaria diagnosis, it is important to acknowledge its limitations. For instance, deploying such technology in the field, where expert microbiologists are scarce, faces significant practical challenges related to budgeting. Despite this challenge, CAM’s contribution to standardizing the evaluation of TBS coloration quality offers a novel approach that complements traditional methods, especially in resource-limited settings where expert microscopists may not be accessible.

## Conclusions

This work successfully engineered a novel aid system named CAM. It evaluates the staining procedures used during sample preparation, offering a new perspective on automated malaria diagnosis that can lead to more accurate and efficient disease detection.

After testing, the 15-bin histogram of the ‘H’ component from the HSV colour space was the most effective feature vector for training CAM. Built on a Cubic SVM classifier, CAM demonstrated a True Negative Rate (TNR) of 95% and a True Positive Rate (TPR) of 97%.

CAM could help ensure accurate malaria diagnosis in remote diagnostic centres, regardless of variations in smear staining. This solution is a supportive tool for analysing the coloration quality of thick blood smears, empowering microscopists to examine each microscopic field carefully when necessary. Moreover, this solution holds potential for external performance evaluations conducted by official laboratories. Microscopist abilities, including smear staining capabilities, are closely monitored and evaluated in these settings. Implementing CAM can facilitate objective assessments that are not subject to evaluation personnel’s visual perception biases, improving accuracy and consistency in malaria diagnosis.

## Supplementary Information

Supplementary Material 1: Table S1. Summary of the TNR, TPR, and F1-score obtained by each classifier during training. Table S2. Rates obtained during the system adjustment. These values were obtained from the validation set using the selected four features. Table S3. Features and parameters that showed the best results using the validation set.

## Data Availability

The dataset generated and analyzed during the current study is available at ResearchGate in the link we provided in the methodology section (10.5281/zenodo.7191424). The database is published on: https://drive.google.com/drive/folders/1Qrv0e4bSEtkeqtPABz-klQp-6D6OjU-X?usp=sharing. The database instructions of the database can be found on: https://www.researchgate.net/publication/359826910_Database600Labels_Instructionstxt. The labels of the database are published on: https://www.researchgate.net/publication/359827216_Database_600Labelscsv.

## Abbreviations

TBS: Thick blood smears
INS-Colombia: Instituto Nacional de Salud de Colombia (National Institute of Health-Colombia)
WHO: World Health Organization
SVM: Support vector machine
TNR: True negative rate
TPR: True positive rate
