# Supplementary material for: CAM: a novel aid system to analyse the coloration quality of thick blood smears using image processing and machine learning techniques
Source: Malar J. 2024 Oct 7;23:299. doi: 10.1186/s12936-024-05025-7 (PMC11459806; doi:10.1186/s12936-024-05025-7)
Supplement: Supplementary file 1 — Supplementary Material 1: Table S1. Summary of the TNR, TPR, and F1-score obtained by each classifier during training. Table S2. Rates obtained during the system adjustment. These values were obtained from the validation set using the selected four features. Table S3. Features and parameters that showed the best results using the validation set. [file 12936_2024_5025_MOESM1_ESM.docx]

Supplementary Materials for

**CAM: A Novel Aid System to Analyze the Coloration Quality of Thick Blood Smears Using Image Processing and Machine Learning Techniques**

W. M. Fong Amarís^1,2*^, Daniel R. Suárez^2^, Liliana J Cortés-Cortés^3^, Carol Martinez^4*^

^1^ Ph.D. candidate in Biotechnology. Universidade Federal do Pará, Belém, Brazil (Programa de Pós-Graduação em Biotecnologia). ^2^ Facultad de Ingeniería, Pontificia Universidad Javeriana, Bogotá, Colombia. ^3^ National Reference Laboratory, Parasitology Group, National Health Institute of Colombia, Bogotá. ^4*^ Research Scientist. Space Robotics Research Group (SpaceR), Interdisciplinary Centre for Security, Reliability, and Trust (SnT), University of Luxembourg. * Corresponding author

Corresponding authors: wendy.amaris@icb.ufpa.br; wfong110@gmail.com; carol.martinezluna@uni.lu

**The PDF file includes:**

***Tables S1 to S3:***

Table S1. Summary of the TNR, TPR, and F1-score obtained by each classifier during training.

Table S2. Rates obtained during the system adjustment. These values were obtained from the validation set using the selected four features.

Table S3 Features and parameters that showed the best results using the validation set.

**Features and classifiers exploration**

**Table S1:** Summary of the TNR, TPR and F1-score obtained by each classifier during the training process.

| **Feature** | **Variables** | **Type of histogram** | **Classifiers results** | | | | | | | | | | | |
| --- | --- | --- | --- | --- | --- | --- | --- | --- | --- | --- | --- | --- | --- | --- |
|  |  |  | **Classifier 1** | **TNR** | **TPR** | **F1-score** | **Classifier 2** | **TNR** | **TPR** | **F1-score** | **Classifier 3** | **TNR** | **TPR** | **F1-score** |
| Specific variables of histogram | H5-S35-V114 | Normalized | Ensemble (Bagged trees) | 0,91 | 0,89 | 0,89 | Ensemble (Boosted trees) | 0,90 | 0,87 | 0,88 | - | - | - | - |
|  |  | Without normalizing | Ensemble (Bagged trees) | 0,90 | 0,89 | 0,89 | Ensemble (Coarse tree) | 0,90 | 0,89 | 0,89 | - | - | - | - |
| Variables from PCA | H1, H2, H3 | **Normalized** | SVM (Quadratic SVM) | 0,99 | 0,96 | 0,97 | SVM (Cubic SVM) | 0,98 | 0,96 | 0,96 | SVM (Medium Gaussian SVM) | 0,99 | 0,96 | 0,97 |
|  |  | Without normalizing | SVM (Quadratic SVM) | 0,99 | 0,98 | 0,98 | SVM (Cubic SVM) | 0,99 | 0,97 | 0,97 | SVM (Coarse Gaussian SVM) | 0,99 | 0,93 | 0,95 |
|  | S1, S2, S3 | Normalized | SVM (Cubic SVM) | 0,91 | 0,81 | 0,85 | SVM (Lineal SVM) | 0,94 | 0,61 | 0,73 | - | - | - | - |
|  |  | Without normalizing | Ensemble (Boosted trees) | 0,92 | 0,92 | 0,92 | KNN (Medium KNN) | 0,92 | 0,89 | 0,90 | - | - | - | - |
|  | HS1, HS2, HS3 | Normalized | KNN (Fine KNN) | 0,98 | 0,97 | 0,96 | KNN (Medium KNN) | 0,99 | 0,95 | 0,96 | KNN (Coarse KNN) | 1 | 0,82 | 0,90 |
|  |  | Without normalizing | Ensemble (Bagged trees) | 0,98 | 0,96 | 0,96 | SVM (Fine Gaussian SVM) | 0,97 | 0,96 | 0,96 | - | - | - | - |
| Variances | H, S, HS | Normalized | Ensemble (RUSBoosted trees) | 0,88 | 0,87 | 0,87 | KNN (Cosine KNN) | 0,88 | 0,75 | 0,80 | - | - | - | - |
|  |  | Without normalizing | KNN (Weighted KNN) | 0,91 | 0,93 | 0,92 | Ensemble (Bagged Trees) | 0,92 | 0,93 | 0,92 | - | - | - | - |
| Correlation coefficients | H, S, HS | **Without normalizing** | SVM (Cubic SMV) | 0,99 | 0,96 | 0,97 | SVM (Fine Gaussian SVM) | 0,99 | 0,94 | 0,95 | SVM (Quadratic SVM) | 0,98 | 0,96 | 0,96 |
| Full histogram values (bins numbers = 15) | H | **Normalized** | SVM (Cubic SVM) | 0,99 | 0,98 | 0,98 | SVM (Quadratic SVM) | 0,99 | 0,97 | 0,97 | - | - | - | - |
|  |  | Without normalizing | Ensemble (Subspace KNN) | 0,98 | 0,99 | 0,98 | Ensemble (Bagged trees) | 0,99 | 0,97 | 0,97 | SVM (Cubic SVM) | 0,99 | 0,97 | 0,97 |
|  | S | Normalized | SVM (Cubic SVM) | 0,91 | 0,84 | 0,92 | - | - | - | - | - | - | - | - |
|  |  | Without normalizing | KNN (Weighted KNN) | 0,92 | 0,93 | 0,92 | - | - | - | - | - | - | - | - |
|  | HS | Normalized | Ensemble (Subspace KNN) | 0,99 | 0,98 | 0,98 | KNN (Fine KNN) | 0,99 | 0,97 | 0,97 | SVM (Cubic SVM) | 0,99 | 0,96 | 0,97 |
|  |  | Without normalizing | Ensemble (Subspace KNN) | 0,99 | 0,98 | 0,98 | SVM (Cubic SVM) | 0,99 | 0,97 | 0,97 | - | - | - | - |
| Variables from PCA (Total bins number = 14) | H1, H2, H3, S1, S2, S3, HS1, HS2, HS3 | **Normalized** | Logistic regression | 0,99 | 0,96 | 0,97 | SVM (Quadratic SVM) | 0,99 | 0,96 | 0,97 | SVM (Cubic SVM)  SVM (Fine Gaussian SVM) | 0,99 | 0,96 | 0,97 |
|  |  | Without normalizing | KNN (Fine KNN) | 0,99 | 0,99 | 0,99 | SVM (Cubic SVM) | 0,99 | 0,97 | 0,97 | SVM (Fine Gaussian SVM) | 0,98 | 0,98 | 0,98 |

**Table S2:** Rates obtained during the system adjustment. These values were obtained from the validation set and using the four features selected (part 1).

| **Features** | **Parameters** | | **Quadratic SVM** | | | | | **Cubic SVM** | | | | |
| --- | --- | --- | --- | --- | --- | --- | --- | --- | --- | --- | --- | --- |
|  |  |  | **Misclassif *** | **TNR**** | **TPR***** | **Accuracy** | **F1 - score** | **Misclassif** | **TNR** | **TPR** | **Accuracy** | **F1 - score** |
| **PCA H1,H2,H3** | **Cross validation** | **3 fold** |  |  |  |  |  |  |  |  |  |  |
|  | Kernel Scale | Default ('auto') | **0,0190** | **0,9778** | **0,9333** | **0,9556** | **0,9130** | **0,0190** | **0,9556** | **0,9333** | **0,9444** | **0,9231** |
|  | Kernel Scale | 6.9 | 0,0357 | 1,0000 | 0,8667 | 0,9333 | 0,8125 | 0,0381 | 1,0000 | 0,9111 | 0,9556 | 0,8723 |
|  | Kernel Scale | 1.7 | 0,0190 | 1,0000 | 0,9111 | 0,9556 | 0,8723 | **0,0167** | **0,9556** | **0,9333** | **0,9444** | **0,9231** |
|  | Kernel Scale | 0.43 | 0,0190 | 0,9333 | 0,9556 | 0,9556 | 0,8723 | 0,0214 | 0,9333 | 0,9333 | 0,9333 | 0,9333 |
|  | **Cross validation** | **5 fold** |  |  |  |  |  |  |  |  |  |  |
|  | Kernel Scale | Default ('auto') | **0,0167** | **0,9778** | **0,9333** | **0,9556** | **0,9130** | **0,0190** | **0,9556** | **0,9333** | **0,9444** | **0,9231** |
|  | Kernel Scale | 6.9 | 0,0333 | 1,0000 | 0,9111 | 0,9556 | 0,8723 | 0,0381 | 1,0000 | 0,9111 | 0,9556 | 0,8723 |
|  | Kernel Scale | 1.7 | 0,0190 | 0,9778 | 0,9333 | 0,9556 | 0,9130 | **0,0167** | **0,9556** | **0,9333** | **0,9444** | **0,9231** |
|  | Kernel Scale | 0.43 | 0,0143 | 0,9556 | 0,9333 | 0,9444 | 0,9231 | 0,0214 | 0,9333 | 0,9333 | 0,9333 | 0,9333 |
|  | **Cross validation** | **10 fold** |  |  |  |  |  |  |  |  |  |  |
|  | Kernel Scale | Default ('auto') | **0,0190** | **0,9778** | **0,9333** | **0,9556** | **0,9130** | 0,0214 | 0,9333 | 0,9333 | 0,9333 | 0,9333 |
|  | Kernel Scale | 6.9 | 0,0333 | 1,0000 | 0,9111 | 0,9556 | 0,8723 | 0,0333 | 1,0000 | 0,9111 | 0,9556 | 0,8723 |
|  | Kernel Scale | 1.7 | 0,0190 | 0,9778 | 0,9111 | 0,9444 | 0,8817 | **0,0167** | **0,9556** | **0,9333** | **0,9444** | **0,9231** |
|  | Kernel Scale | 0.43 | 0,0190 | 0,9333 | 0,9556 | 0,9444 | 0,9663 | 0,0190 | 0,9333 | 0,9556 | 0,9444 | 0,9663 |
| **Correlation coefficients (H,S,HS)** | **Cross validation** | **3 fold** |  |  |  |  |  |  |  |  |  |  |
|  | Kernel Scale | Default ('auto') | 0,0381 | 0,9778 | 0,8889 | 0,9333 | 0,8511 | **0,0238** | **0,9778** | **0,9333** | **0,9556** | **0,9130** |
|  | Kernel Scale | 6.9 | 0,0548 | 0,9778 | 0,8222 | 0,9000 | 0,7626 | 0,0571 | 0,9778 | 0,8222 | 0,9000 | 0,7626 |
|  | Kernel Scale | 1.7 | 0,0452 | 0,9778 | 0,8889 | 0,9333 | 0,8511 | 0,0429 | 0,9778 | 0,8889 | 0,9333 | 0,8511 |
|  | Kernel Scale | 0.43 | **0,0190** | **0,9778** | **0,9333** | **0,9556** | **0,9130** | **0,0286** | **0,9556** | **0,9333** | **0,9444** | **0,9231** |
|  | **Cross validation** | **5 fold** |  |  |  |  |  |  |  |  |  |  |
|  | Kernel Scale | Default ('auto') | 0,0310 | 0,9778 | 0,9111 | 0,9444 | 0,8817 | 0,0238 | 0,9778 | 0,8889 | 0,9333 | 0,8511 |
|  | Kernel Scale | 6.9 | 0,0548 | 0,9778 | 0,8222 | 0,9000 | 0,7629 | 0,0571 | 0,9778 | 0,8444 | 0,9111 | 0,7917 |
|  | Kernel Scale | 1.7 | 0,0524 | 0,9778 | 0,9111 | 0,9444 | 0,8817 | 0,0310 | 0,9556 | 0,8889 | 0,9222 | 0,8602 |
|  | Kernel Scale | 0.43 | **0,0333** | **0,9778** | **0,9333** | **0,9556** | **0,9130** | **0,0214** | **0,9778** | **0,9333** | **0,9556** | **0,9130** |
|  | **Cross validation** | **10 fold** |  |  |  |  |  |  |  |  |  |  |
|  | Kernel Scale | Default ('auto') | 0,0333 | 0,9778 | 0,8889 | 0,9333 | 0,8511 | 0,0190 | 0,9778 | 0,9111 | 0,9444 | 0,8817 |
|  | Kernel Scale | 6.9 | 0,0595 | 0,9778 | 0,8222 | 0,9000 | 0,7629 | 0,0595 | 0,9778 | 0,8444 | 0,9111 | 0,7917 |
|  | Kernel Scale | 1.7 | 0,0476 | 0,9778 | 0,9111 | 0,9444 | 0,8817 | 0,0310 | 0,9556 | 0,8889 | 0,9222 | 0,8602 |
|  | Kernel Scale | 0.43 | 0,0238 | 0,9778 | 0,8889 | 0,9333 | 0,8511 | 0,0190 | 0,9778 | 0,9111 | 0,9444 | 0,8817 |

*Misclassif: Misclassification rate **TNR: True Negative Rate ***TPR: True Positive Rate

**Table S2:** Rates obtained during the system adjustment. These values were obtained from the validation set and using the four features selected (part 2).

| **Features** | **Parameters** | | **Quadratic SVM** | | | | | **Cubic SVM** | | | | |
| --- | --- | --- | --- | --- | --- | --- | --- | --- | --- | --- | --- | --- |
|  |  |  | **Misclassif** | **TNR** | **TPR** | **Accuracy** | **F1 - score** | **Misclassif** | **TNR** | **TPR** | **Accuracy** | **F1 - score** |
| **Full features vectors (H)** | **Cross validation** | **3 fold** |  |  |  |  |  |  |  |  |  |  |
|  | Kernel Scale | Default ('auto') | **0,0190** | **0,9778** | **0,9556** | **0,9667** | **0,9451** | 0,0143 | 0,9778 | 0,9333 | 0,9556 | 0,9130 |
|  | Kernel Scale | 6.9 | 0,0214 | 0,9778 | 0,9556 | 0,9667 | 0,9451 | **0,0238** | **0,9778** | **0,9556** | **0,9667** | **0,9451** |
|  | Kernel Scale | 1.7 | 0,0143 | 0,9556 | 0,9778 | 0,9667 | 0,9888 | 0,0190 | 0,9778 | 0,9333 | 0,9556 | 0,9130 |
|  | Kernel Scale | 0.43 | 0,0143 | 0,9556 | 0,9778 | 0,9667 | 0,9888 | 0,0286 | 0,9556 | 1,0000 | 0,9778 | 1,0000 |
|  | **Cross validation** | **5 fold** |  |  |  |  |  |  |  |  |  |  |
|  | Kernel Scale | Default ('auto') | 0,0190 | 0,9556 | 0,9556 | 0,9556 | 0,9556 | 0,0238 | 0,9556 | 0,9778 | 0,9667 | 0,9888 |
|  | Kernel Scale | 6.9 | **0,0238** | **0,9778** | **0,9556** | **0,9667** | **0,9451** | **0,0190** | **0,9778** | **0,9556** | **0,9667** | **0,9451** |
|  | Kernel Scale | 1.7 | 0,0095 | 0,9556 | 0,9778 | 0,9667 | 0,9888 | 0,0167 | 0,9778 | 0,9333 | 0,9556 | 0,9130 |
|  | Kernel Scale | 0.43 | 0,0167 | 0,9556 | 0,9556 | 0,9556 | 0,9556 | 0,0143 | 0,9111 | 0,9556 | 0,9333 | 0,9773 |
|  | **Cross validation** | **10 fold** |  |  |  |  |  |  |  |  |  |  |
|  | Kernel Scale | Default ('auto') | 0,0190 | 0,9556 | 0,9556 | 0,9556 | 0,9556 | 0,0143 | 0,9556 | 0,9778 | 0,9667 | 0,9888 |
|  | Kernel Scale | 6.9 | **0,0238** | **0,9778** | **0,9556** | **0,9667** | **0,9451** | **0,0190** | **0,9778** | **0,9556** | **0,9667** | **0,9451** |
|  | Kernel Scale | 1.7 | 0,0143 | 0,9556 | 0,9778 | 0,9667 | 0,9888 | 0,0119 | 0,9778 | 0,9333 | 0,9556 | 0,9130 |
|  | Kernel Scale | 0.43 | 0,0119 | 0,9556 | 0,9778 | 0,9667 | 0,9888 | 0,0143 | 0,9556 | 0,9333 | 0,9444 | 0,9231 |
| **PCA**  **(H1, H2, H3,**  **S1, S2, S3,**  **HS1, HS2,**  **HS3)** | **Cross validation** | **3 fold** |  |  |  |  |  |  |  |  |  |  |
|  | Kernel Scale | Default ('auto') | 0,0238 | 1,0000 | 0,2222 | 0,6111 | 0,1600 | 0,0190 | 0,0000 | 1,0000 | 0,5000 | NaN |
|  | Kernel Scale | 6.9 | 0,0381 | 0,0000 | 1,0000 | 0,5000 | NaN | 0,0381 | 0,0000 | 1,0000 | 0,5000 | NaN |
|  | Kernel Scale | 1.7 | 0,0190 | 0,9111 | 0,1110 | 0,5111 | 0,0794 | 0,0190 | 0,0000 | 1,0000 | 0,5000 | NaN |
|  | Kernel Scale | 0.43 | 0,0381 | 0,9778 | 0,2444 | 0,6111 | 0,1789 | 0,0262 | 0,0000 | 1,0000 | 0,5000 | NaN |
|  | **Cross validation** | **5 fold** |  |  |  |  |  |  |  |  |  |  |
|  | Kernel Scale | Default ('auto') | 0,0214 | 0,8889 | 0,4444 | 0,6667 | 0,3636 | 0,0190 | 0,0000 | 1,0000 | 0,5000 | NaN |
|  | Kernel Scale | 6.9 | 0,0381 | 0,0000 | 1,0000 | 0,5000 | NaN | 0,0357 | 0,0000 | 1,0000 | 0,5000 | NaN |
|  | Kernel Scale | 1.7 | 0,0190 | 1,0000 | 0,2889 | 0,6444 | 0,2131 | 0,0190 | 0,0000 | 1,0000 | 0,5000 | NaN |
|  | Kernel Scale | 0.43 | 0,0262 | 0,9556 | 0,1333 | 0,5444 | 0,0945 | 0,0310 | 0,0000 | 1,0000 | 0,5000 | NaN |
|  | **Cross validation** | **10 fold** |  |  |  |  |  |  |  |  |  |  |
|  | Kernel Scale | Default ('auto') | 0,0238 | 1,0000 | 0,4444 | 0,7222 | 0,3478 | 0,0190 | 0,0000 | 1,0000 | 0,5000 | NaN |
|  | Kernel Scale | 6.9 | 0,0381 | 0,0000 | 1,0000 | 0,5000 | NaN | 0,0333 | 0,0000 | 1,0000 | 0,5000 | NaN |
|  | Kernel Scale | 1.7 | 0,0190 | 1,0000 | 0,1778 | 0,5889 | 0,1260 | 0,0167 | 0,0000 | 1,0000 | 0,5000 | NaN |
|  | Kernel Scale | 0.43 | 0,0310 | 0,9111 | 0,1333 | 0,5222 | 0,0960 | 0,0238 | 0,0000 | 1,0000 | 0,5000 | NaN |

*Misclassif: Misclassification rate **TNR: True Negative Rate ***TPR: True Positive Rate

**Classifier Optimization**

Finally, two types of parameters were modified:

1. The number of folds: 3, 5, and 10 folds. These variations were selected based on previous studies by Rahman *et al.* in 2019 [25] and Das *et al.* in 2014 [44].
2. Kernel scale: We evaluated the following values: 0.43, 1.7, and 6.9. Each one was randomly selected.

With these values, the Quadratic and Cubic SVM classifiers were trained (with the training set), and the results were evaluated using our dataset's validation set. The best classifier and the best feature vector were evaluated using the following metrics: the misclassification rate, TPR, TNR, accuracy, and F1-score.

We found that the feature vector 4 [H_PCA1,_ H_PCA2,_ H_PCA3,_ S_PCA1,_ S_PCA2,_ S_PCA3,_ HS_PCA1,_ HS_PCA2,_ HS_PCA3_] did not show good results in any of the metrics (Table 2 in supplementary material). For that reason, this feature was discarded as a candidate feature to evaluate the coloration quality in thick blood smears.

**Table S3** presents the best results obtained by each classifier and shows the parameters responsible for those rates. Because the classification rates were similar among the classifiers when the TNR values were considered, the F1-score and misclassification rate were the metrics used to choose the classifiers and features. The best algorithms obtained values > 92% and 94%. Based on these values, feature vector 2 (H_corr_, S_corr_, HS_corr_) with an F1-score of 91% was discarded.

Based on the above, the best features to analyze the coloration quality in thick blood smear images are features vectors 1 [HPCA1, HPCA2, HPCA3] and 3 (Hist15_H), the ones shown in Table S3 in bold letters.

**Table S3** Features and parameters that showed the best results using the validation set.

| **Features vector** | **Parameters** | | **SVM** | **Rates** | | | | |
| --- | --- | --- | --- | --- | --- | --- | --- | --- |
|  |  |  |  | **Misclassif*** | **TNR**** | **TPR ***** | **Accuracy** | **F1-score** |
| 1. [H_PCA1,_  H_PCA2,_  H_PCA3_] | Cross validation | 5 fold | Quadratic | 0,0167 | 0,9778 | 0,9333 | 0,9556 | 0,9130 |
|  | Kernel Scale | Default |  |  |  |  |  |  |
|  | Cross validation | 5 fold | Cubic | **0,0167** | **0,9556** | **0,9333** | **0,9444** | **0,9231** |
|  | Kernel Scale | 1.7 |  |  |  |  |  |  |
| 2. [H_corr,_  S_corr,_  HS_corr_] | Cross validation | 3 fold | Quadratic | 0,0190 | 0,9778 | 0,9333 | 0,9556 | 0,9130 |
|  | Kernel Scale | 0.43 |  |  |  |  |  |  |
|  | Cross validation | 5 fold | Cubic | 0,0214 | 0,9778 | 0,9333 | 0,9556 | 0,9130 |
|  | Kernel Scale | 0.43 |  |  |  |  |  |  |
| 3. Hist_15__H | Cross validation | 3 fold | Quadratic | 0,0190 | 0,9778 | 0,9556 | 0,9667 | 0,9451 |
|  | Kernel Scale | Default |  |  |  |  |  |  |
|  | Cross-validation | 5 fold | Cubic | **0,0190** | **0,9778** | **0,9556** | **0,9667** | **0,9451** |
|  | Kernel Scale | 6.9 |  |  |  |  |  |  |

*Misclassif: Misclassification rate **TNR: True Negative Rate ***TPR: True Positive Rate

Although the misclassification rate is low for feature vector 1 [H_PCA1_, H_PCA2_, H_PCA3_], feature vector 3 (Hist_15__H) showed the best TNR rate. Additionally, because the best results were obtained with the Cubic SVM, this classifier was selected to conduct the final analysis, employing the test set of the database.
